# Cardiorenal Syndrome is Present in Human Fetuses with Severe, Isolated Urinary Tract Malformations

**DOI:** 10.1371/journal.pone.0063664

**Published:** 2013-05-22

**Authors:** Waltraut M. Merz, Kirsten Kübler, Rolf Fimmers, Arne Willruth, Birgit Stoffel-Wagner, Ulrich Gembruch

**Affiliations:** 1 Department of Obstetrics and Prenatal Medicine, University of Bonn Medical School, Bonn, Germany; 2 Institute for Medical Biometry, Informatics and Epidemiology, University of Bonn Medical School, Bonn, Germany; 3 Institute for Clinical Chemistry and Pharmacology, University of Bonn Medical School, Bonn, Germany; Université de Montréal, Canada

## Abstract

**Objective:**

We analyzed the association between renal and cardiovascular parameters in fetuses with isolated severe urinary tract malformations.

**Methods:**

39 fetuses at a mean gestational age of 23.6 weeks with nephropathies or urinary tract malformations and markedly impaired or absent renal function were prospectively examined. Fetal echocardiography was performed, and thicknesses of the interventricular septum, and left and right ventricular wall were measured. Blood flow velocity waveforms of the umbilical artery, middle cerebral artery, and ductus venosus were obtained by color Doppler ultrasound. Concentrations of circulating n-terminal pro-B-type natriuretic peptide (nt-proBNP), cystatin C, ß_2_-microglobulin, and hemoglobin were determined from fetal blood samples.

**Results:**

Malformations included 21 cases of obstructive uropathy, 10 fetuses with bilateral nephropathy, and 8 cases of bilateral renal agenesis. Marked biventricular myocardial hypertrophy was present in all cases. The ratio between measured and gestational age-adjusted normal values was 2.01 (interventricular septum), 1.85, and 1.78 (right and left ventricular wall, respectively). Compared to controls, levels of circulating nt-proBNP were significantly increased (median (IQR) 5035 ng/L (5936 ng/L) vs. 1874 ng/L (1092 ng/L); p<0.001). Cystatin C and ß_2_-microglobulin concentrations were elevated as follows (mean ± SD) 1.85±0.391 mg/L and 8.44±2.423 mg/L, respectively (normal range 1.66±0.202 mg/L and 4.25±0.734 mg/L, respectively). No correlation was detected between cardiovascular parameters and urinary tract morphology and function. Despite increased levels of nt-proBNP cardiovascular function was preserved, with normal fetal Doppler indices in 90.2% of cases.

**Conclusion:**

Urinary tract malformations resulting in severe renal impairment are associated with biventricular myocardial hypertrophy and elevated concentrations of circulating nt-proBNP during fetal life. Cardiovascular findings do not correlate with kidney function or morphology.

## Introduction

In children and adults chronic kidney disease (CKD) is associated with an increased rate of cardiovascular complications [1], [2], [3]. Traditional risk factors like hypertension, dyslipidemia, obesity and alterations in glucose metabolism as well as uremia-related factors including volume overload, anemia, abnormal calcium and phosphate metabolism, malnutrition, and inflammation contribute to the development of the various forms of the cardiorenal syndrome (CRS) [1], [4]. Accelerated atherosclerosis and left-ventricular hypertrophy with diastolic and systolic dysfunction are present already in children with CKD [4], [5].

In adult and pediatric cardiology n-terminal pro-B-type natriuretic peptide (nt-proBNP), the inactive cleavage product of brain natriuretic peptide (BNP) is an established marker of cardiovascular dysfunction [6], [7]. Increased levels of circulating nt-proBNP are the result of re-expression of the cardiac embryonic gene program and induce cardiac remodeling and fibrosis [8], [9]. During human development the cardiac natriuretic peptide system, comprising atrial natriuretic peptide (ANP) and BNP is functional by mid-gestation, and nt-proBNP concentrations have been found to be elevated in conditions associated with increased volume or pressure load [10]–[16].

The aim of our study was to analyze the association between renal and cardiovascular parameters in fetuses with severely reduced or absent renal function secondary to isolated urinary tract malformations. For that, nt-proBNP, cystatin C, ß_2_-microglobulin, and hemoglobin was analyzed in umbilical vein samples of affected fetuses. Additionally, echocardiography and color Doppler examination of the fetal cardiovascular system was performed, and myocardial wall dimensions and cardiothoracic area were measured.

Our hypothesis was that renal and cardiac function is interrelated during gestation, and that changes in cardiovascular function are present in fetuses with impaired or absent renal function.

## Materials and Methods

### Patients

Patients referred to our center between January 2007 and October 2011 with a diagnosis of bilateral nephropathy or urinary tract malformation who underwent fetocide or fetal blood sampling (FBS) were eligible. Fetocide was performed as part of termination of advanced pregnancy in accordance with national legislation. Indications for FBS included rapid karyotyping due to late referral or kidney function analysis. We excluded fetuses with any of the following conditions: unilateral nephropathy, unilateral urinary tract malformation, associated structural malformations, aneuploidy, moderate or severe anemia [17], monochorionic twin pregnancies, and cases with intrauterine growth restriction (estimated fetal birth weight <10. percentile). Gestational age (GA) was confirmed by first-trimester ultrasound. For nt-proBNP analysis, previously established reference values served as controls. Details of the control group have already been published [16].

### Ethics Statement

The study was approved by the university of Bonn ethics committee. All participating pregnant women gave their written consent.

### Ultrasonograhy and Echocardiography

High-resolution ultrasound equipment (Philips iu22, Philips Hamburg, Germany; GE logiq 9, Voluson E8, Voluson730 expert, GE Munich, Germany) was used in all cases with 5 to 9 and 2 to 6, 7, and 8 MHz respectively, convex transducers. The spatial peak temporal average power output was kept at <100 W/cm^2^, applying only the fetal use adapted ultrasound machine settings. A detailed ultrasound of the fetal anatomy, echocardiography with measurement of myocardial wall dimensions, and color Doppler examination of the fetal and uterine vessels was performed. Thicknesses of the interventricular septum (IVS) and the right (RVW) and left (RVW) ventricular wall were determined in B-mode; a standard lateral four-chamber-view was obtained, and measurements were taken at the tip of the atrioventricular valve leaflets during diastole. Doppler recordings of blood flow velocities in the middle cerebral artery (MCA) and umbilical artery (UA) were obtained at an insonation angle between 0° and maximally 10° to flow and <30° for ductus venosus (DV) and uterine arteries (utA), using standard positioning of the sample volume; angle correction was not performed. The high-pass-filter was set at 60 Hz. At least five consecutive uniform Doppler velocity waveforms with the highest velocities and a narrow band of frequencies were recorded, and one cycle was analyzed. Indices were calculated accordingly; abnormal values were defined as UA pulsatility index >90th percentile for GA or absent or reversed end-diastolic flow; DV pulsatility index for veins >90th percentile for GA or absent or negative a-wave; utA resistance index >90th percentile for GA or bilateral notching; peak systolic velocity of the middle cerebral artery (PSV-MCA) >1.28 times the median for GA. All examinations were performed prior to invasive intervention.

### Sample Collection and Processing

Specimens were collected as part of the FBS procedure (n = 29, 70.7%) or fetocide (n = 12, 29.3%). The umbilical cord was punctured under ultrasound control near its placental insertion site. One mL of fetal venous blood was withdrawn either before injecting the cardioplegic solution or before proceeding with the FBS.

NT-proBNP was measured by chemiluminescence immunoassay, cystatin C and ß_2_-microglobulin were analyzed by nephelometry, all on a Dimension Vista 1500 (Siemens Healthcare Diagnostics, Eschborn, Germany). Hemoglobin was analyzed on a Sysmex XE-2100 (Sysmex, Norderstedt, Germany). Inter- and intra-assay coefficients of variation were 3.5% and 2.3% for nt-proBNP, 2.6% and 2.4% for cystatin C, 3.2% and 3.1% for ß_2_-microglobulin, and 0.41% and 0.38% for hemoglobin. All samples were processed within two hours after retrieval.

Vesico-amniotic shunting was performed under ultrasound control. Using Seldinger technique a Harrison fetal bladder stent 5.0 Fr/1.3-3.5 cm (Cook Medical Technology Park, Casletroy Limerick, Republic of Ireland) was inserted under local anesthesia. Correct position was verified by ultrasound and indirectly by prompt reduction in bladder volume.

### Statistical Analysis

To adjust for the effect of gestational age hemoglobin values were transformed into multiples of the median (MoM). Expected hemoglobin values were calculated with the following formula: e^(2.84–8.55/GA)^ [17]. MoMs were then calculated by dividing the measured values by the expected values. Anemia was classified as mild (0.84–0.65 MoM), moderate (0.64–0.55 MoM), and severe (<0.55 MoM) [17].

Gestational age-adjusted normal values and standard deviations were calculated for the interventricular septum (IVS-GA) and the right and left ventricular wall (RVW-GA and LVW-GA) using the following formulas: IVS-GA = −0.1321+(0.01927 * GA)−(0.00018 * GA^2^); RVW-GA = −0.1473+(0.02045 * GA)−(0.0002 * GA^2^); LVW-GA = −0.136+(0.01967 * GA)−(0.00019 * GA^2^) [18]. Ratios ware calculated by dividing measured from normal values.

For normally distributed values, between group comparisons of continuous variables were performed by independent samples t-test or one-way ANOVA with post-hoc analysis by Tamhane-T2 Test. For nt-proBNP and IVS, Mann-Whitney-U or Kruksal-Wallis test was used. Categorical variables were compared by χ^2^ test. For correlation analysis Pearson’s coefficient was calculated. Unless indicated otherwise, results are reported as mean ± SD.

## Results

41 analyses were performed in 39 fetuses. In two fetuses with obstructive uropathy measurements were taken twice (before and after shunting) with two weeks interval. Cystatin C and hemoglobin values could not be determined in three cases, ß_2_-microglobulin in two. Mean gestational age was 23.6 (±3.57 weeks of gestation), 30 fetuses were male (76.9%). A normal karyotype was present in all examined cases (n = 36, 92.3%).

Malformations were classified in three categories:

1. Obstructive uropathy, n = 21 (53.9%): lower (urethral) urinary tract obstruction with or without development of urinoma or urinous ascites.
2. Bilateral nephropathy, n = 10 (25.6%): autosomal recessive polycystic kidney disease; bilateral multicystic dysplastic kidneys; unilateral multicystic dysplastic kidney with contralateral renal agenesis or dysplasia.
3. Bilateral renal agenesis, n = 8 (20.5%).

Severe oligohydramnios (amniotic fluid index ≤2 cm) or anhydramnios was present in all cases. Pulmonary hypoplasia, defined as cardiothoracic area ratio (CTAR) >0.3 could be demonstrated in 36 fetuses (87.8%); CTAR was normal in 3 (7.3%), and measurement was not available for 2 cases (4.9%).

**Figure 1:**
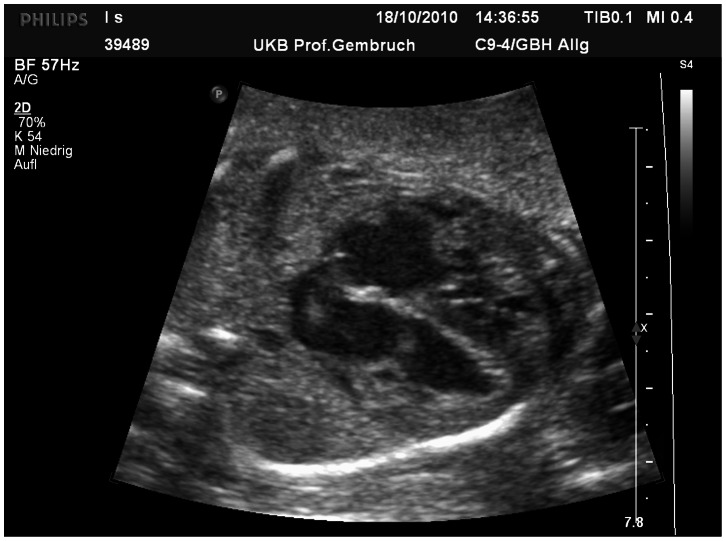
Cardiac findings in urinary tract malformation. Biventricular myocardial hypertrophy in a fetus with bilateral renal agenesis at 21 weeks of gestation.

Myocardial wall dimensions were markedly altered, see Figure 1 and Table 1. No correlation was detected between myocardial wall thicknesses and any of the serological parameters under investigation, and no difference was present between the malformation groups. Qualitative echocardiography revealed no signs of cardiac failure in any of the fetuses. Pulsed wave Doppler sonography of fetal vessels was normal in 90.2% of cases (n = 37). Three fetuses had abnormal DV pulsatility indices (including one case with abnormal PSV-MCA), in one case PSV-MCA only was abnormal. 29 pregnancies (74.4%) were terminated, five (12.8%) ended in live births, and five were lost to follow-up.

**Table 1:** Cardiac dimensions in fetuses with urinary tract malformations (n = 37) and gestational age-adjusted normal values.

	Thickness (mm), mean ± SD	Adjusted normal value (mm), mean ± SD	Ratio^a^, mean ± SD
Interventricular septum	4.30±1.163	2.14±0.643	2.01±0.372
Right ventricular wall	3.97±1.055	2.15±0.597	1.85±0.333
Left ventricular wall	3.78±0.943	2.14±0.570	1.78±0.342

a measured value/gestational age-adjusted normal value.

**Figure 2:**
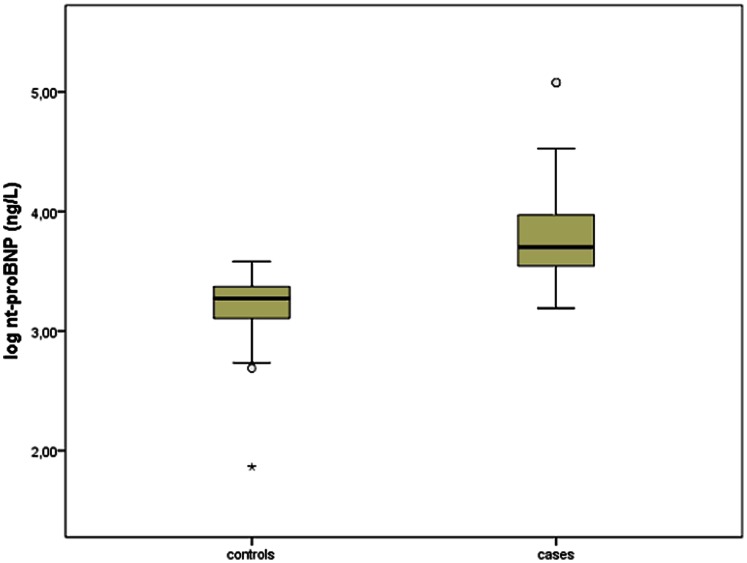
Nt-proBNP concentration in fetuses with urinary tract malformations and controls [16]. Depicted values are log-transformed. *^,^°denotes outliers. Mann-Whitney-U test: p<0.001.

Compared to controls, nt-proBNP concentrations were significantly increased in the study group (median (IQR) 5035 ng/L (5936 ng/L) vs. 1874 ng/L (1092 ng/L), p<0.001), see Figure 2; no difference was present between the three malformation categories. Cystatin C, ß_2_-microglobulin and GA-adapted hemoglobin concentrations are listed in Table 2. Compared to fetal reference values [19], [20] cystatin C and ß_2_-microglobulin were found to be increased. Additionally, cystatin C and ß_2_-microglobulin concentrations were significantly higher in bilateral renal agenesis compared to obstructive uropathies (p = 0.005 and p<0.001, respectively).

**Table 2:** Umbilical vein concentrations of cardiac and renal function tests in fetuses with isolated urinary tract malformations.

	Fetal referencevalues	Studygroup	Obstructiveuropathy^a^	Bilateralnephropathy^a^	Bilateral renalagenesis	
nt-proBNP (ng/L),median (IQR) (n = 41)	1874 (1092)^b^	5035 (5936)	4854 (5994)	5686 (8279)	4872 (4084)	p<0.001^c^
Cystatin C (mg/L),mean ± SD (n = 38)	1.66±0.202	1.84±0.391	1.67±0.350	1.93±0.354	2.18±0.299	p = 0.005^d^
ß_2_-Microglobulin (mg/L),mean ± SD (n = 39)	4.25±0.734	8.44±2.423	7.55±2.127	8.45±2.640	10.79±1.133	p<0.001^d^
MoM Hb^e^ (n = 38)	0.84–1.16	0.95±0.101	0.96±0.115	0.95±0.084	0.93±0.079	

a see text for details;

b 78 cases;

c study group vs. reference values (Mann-Whitney-U test);

d bilateral renal agenesis vs. obstructive uropathy (Tamhane post-hoc test);

e multiples of the median: 5.–95. percentile.

No correlation was present between nt-proBNP and markers of kidney function or hemoglobin, whereas cystatin C and ß_2_-microglobulin concentrations were closely correlated (r = 0.813, p<0.001).

**Figure 3:**
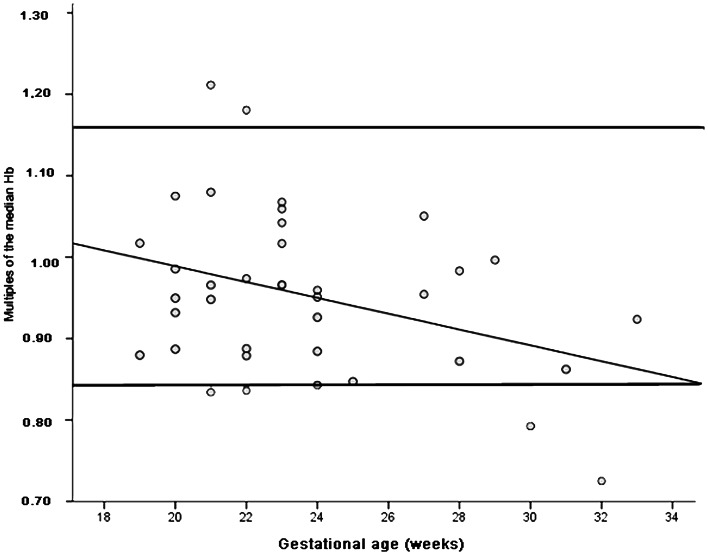
Hemoglobin concentration in fetuses with urinary tract malformations. Multiples of the median hemoglobin (MoM Hb) during the course of pregnancy in fetuses with isolated malformations of the urinary tract (n = 38). Horizontal lines: Upper and lower limit, reference range [17]. R^2^ = 0.127. Equation for the line: y = 1.183–0.010 x GA (gestational age).

A significant inverse correlation between MoM Hb and GA (r = −0.357, p = 0.028) was detected, see Figure 3. Mild anemia was present in 5 cases (13.2%); anemic fetuses did not show any difference in the variables under investigation.

In cases with obstructive uropathy, 14 samples were taken before and nine after vesico-amniotic shunting. There was no difference in any of the variables except for hemoglobin, which was significantly higher after shunting (MoM Hb before versus after shunting: p = 0.010).

## Discussion

In our group of fetuses with severely impaired or absent renal function secondary to isolated nephropathies and urinary tract malformations biventricular myocardial hypertrophy with preserved myocardial function was present. Nt-proBNP concentrations were significantly elevated in the study group compared to controls; no difference was present between the various malformation categories.

### Nt-proBNP during Fetal Life

During human development, natriuretic peptides (NPs) are involved in cardiac morphogenesis. By mid-gestation the cardiac natriuretic system (ANP and BNP) is assumed to be functional. Fetal and maternal compartments are separated and placental transfer of NPs does not occur [10], [21]. In contrast to cardiac morphogenesis NPs are not contributing to renal development. Knock-out mice for natriuretic peptide receptor-A, the principal receptor for atrial natriuretic peptide (ANP) or ANP do not display renal anomalies [22]. The role of nt-proBNP as a marker of increased myocardial work load during fetal life has been investigated in intrauterine growth restriction, structural cardiac malformations, and anemia. Both, increased pressure load secondary to high resistance in the placental bed or outflow tract obstructions and increased volume load secondary to anemia are able to trigger nt-proBNP release [11]–[16]. The longer half-life and higher stability make nt-proBNP a more suitable candidate for analysis compared to BNP [23].

### Myocardial Hypertrophy and Urinary Tract Anomalies during Fetal Life

Limited data are available on the association of myocardial hypertrophy and structural or functional abnormalities of the urinary tract during fetal life. In a series of 50 cases with cardiomyopathy 10% were associated with renal abnormalities [24]. Pedra et al. 2002 [25] in their analysis however did not find nephropathies or urinary tract malformations in association with fetal cardiomyopathy (55 cases). Although levels of circulating nt-proBNP were elevated in our study group no correlation was present between blood levels and myocardial wall thicknesses. Our results therefore do not support involvement of BNP in the development of ventricular wall hypertrophy. Cardiomyocyte hypertrophy was detected in transgenic rats expressing excessive amounts of prorenin [26], and transcription profiling of prorenin-stimulated cardiomyocytes revealed expression of genes involved in actin-filament modification [27]. Further studies are needed to clarify this issue.

### Nt-proBNP and Cardiorenal Syndrome (CRS) in Postnatal Life

The role of nt-proBNP as marker of cardiovascular dysfunction in CKD has been a matter of debate [28]. Meanwhile, it has been proven that cardiac stimulators contribute independently to BNP release in CKD; despite impaired renal clearance nt-proBNP concentrations in CKD are predominantly related to cardiac pathology [29]. Furthermore, correlation between nt-proBNP and myocardial dysfunction has been demonstrated in children with CKD [30]. Nt-proBNP, therefore, serves as a marker of cardiorenal burden in pediatric and adult medicine.

In contrast, the increase in circulating nt-proBNP observed in our study group was not associated with major changes in the fetal cardiovascular function. Therefore, our results do not support a role of nt-proBNP as marker of cardiovascular impairment in nephropathies or urinary tract malformations during fetal life.

### Nt-proBNP and Urinary Tract Anomalies during Fetal Life

Various factors may contribute to the increase in nt-proBNP concentration in fetuses with nephropathies and urinary tract malformations.

Firstly, alterations in the vascular bed of the urinary tract may cause increased afterload.

Renal blood flow in the fetus amounts to 3% of the combined cardiac output and is characterized by high vascular resistance. Vascular resistance within the often grossly enlarged kidneys of severe nephropathy has not been measured. In fetuses with lower urinary tract obstruction increased downstream impedance in the common iliac arteries has been demonstrated and was interpreted to be caused by compression of the distended bladder [31]. Additionally, interstitial fibrosis and hydronephrosis accompanying obstructive uropathy may be associated with increased renovascular resistance.

Secondly, impaired or absent kidney function may result in volume load. In fetal lambs, intra- and extracellular volume is reduced after second-trimester nephrectomy [32]; the same, however, may not apply for nephropathies and urinary tract malformations during fetal life. Postnatally, end-stage renal disease is associated with volume retention which correlates with increasing BNP-levels [33]. During intrauterine life the placenta acts as clearing organ for fetal waste products and is involved in the fluid balance of fetal compartments and amniotic fluid. The distribution of total body water (TBW) in the fetus is subject to dynamic changes; parallel to the reduction in extracellular and increase in intracellular water the TBW to weight ratio decreases. Furthermore, transcapillary fluid filtration into the interstitial space is facilitated during fetal life. Despite placental function these factors may result in a net increase in total body water in fetuses with restricted or absent renal function.

Thirdly, even minor degrees of anemia are able to induce a rise in nt-proBNP [34]. Although moderate or severe anemia was not present in our case series, we observed a significant reduction in hemoglobin with advancing gestational age; additionally, hemoglobin levels were higher after vesico-amniotic shunting.

### Limitations

The number of cases is small, thereby limiting the significance of our findings. Nevertheless, the study represents the largest such cohort in the literature to date. Bökenkamp et al. 2001 [20] and Berry et al. 1995 [35] reported on 20 and 15 fetuses, respectively. Confounding may have been introduced by selection bias. However, all major urinary tract malformation classes were included, and minor or unilateral anomalies and cases with associated malformations excluded. We are therefore confident that our findings are valid. We did not perform quantitative echocardiography and may have missed early signs of myocardial dysfunction. Since qualitative echocardiography and Doppler waveform analyses of fetal vessels including precordial veins were within normal range, no case with major myocardial dysfunction was overlooked. For cardiac dimension calculations we would have preferred to utilize Z-scores [36]. However, nomograms for the parameters under investigation have not been established. Since the measurements in our study group are markedly abnormal we assume our finding to be valid.

### Conclusion

Nephropathies and urinary tract malformations are associated with biventricular myocardial hypertrophy and elevated levels of circulating nt-proBNP during fetal life. The cardiovascular findings do not correlate with kidney function or morphology, but may be caused by changes in prorenin concentrations and increased afterload.

## Supporting Information

Dataset S1 (SAV)

## References

[1] Ronco C, Haapio M, House AA, Anavekar N, Bellomo R (2008) Cardiorenal syndrome. J Am Coll Cardiol 52: 1527–1539. 19007588 10.1016/j.jacc.2008.07.051

[2] Wilson AC, Mitsnefes MM (2009) Cardiovascular disease in CKD in children: Update on risk factors, risk assessment, and management. Am J Kidney Dis 54: 345–360. 19619845 10.1053/j.ajkd.2009.04.027 PMC2714283

[3] Bock JS, Gottlieb SS (2010) Cardiorenal syndrome: new perspectives. Circulation 121: 2592–2600. 20547939 10.1161/CIRCULATIONAHA.109.886473

[4] Patel HP (2010) Early origins of cardiovascular disease in pediatric chronic kidney disease. Renal Failure 32: 1–9. 20113258 10.3109/08860220903288534

[5] Copelovitch L, Warady BA, Furth SL (2011) Insights from the chronic kidney disease in children (CKiD) study. Clin J Am Soc Nephrol 6: 2047–2053. 21784815 10.2215/CJN.10751210 PMC4898858

[6] Hunt SA, Abraham WT, Chin MH, Feldman AM, Francis GS, et al (2005) ACC/AHA 2005 Guideline update for the diagnosis and management of chronic heart failure in the adult – summary article: A report of the American College of Cardiology/American Heart Association Task Force on Practice Guidelines. Circulation 112: 1825–1852.

[7] Dickstein K, Cohen-Solal A, Filippatos G, McMurray JJV, Ponikowski P, et al (2008) ESC Guidelines for the diagnosis and treatment of acute and chronic heart failure 2008. Eur Heart J 29: 2388–2442. 18799522 10.1093/eurheartj/ehn309

[8] McGrath MF, de Bold AJ (2005) Determinants of natriuretic peptide gene expression. Peptides 26: 933–943. 15911063 10.1016/j.peptides.2004.12.022

[9] Potter LR, Yoder AR, Flora DR, Antos LK, Dickey DM (2009) Natriuretic peptides: Their structures, receptors, physiologic functions and therapeutic applications. Handb Exp Pharmacol 191: 341–366. 10.1007/978-3-540-68964-5_15 PMC4855512 19089336

[10] Cameron VA, Ellmers LJ (2003) Minireview: Natriuretic peptides during development of the fetal heart and circulation. Endocrinology 144: 2191–2194. 12746273 10.1210/en.2003-0127

[11] Girsen A, Ala-Kopsala M, Mäkikallio K, Vuolteenaho O, Räsänen J (2007) Cardiovascular hemodynamics and umbilical artery N-terminal peptide of proB-type natriuretic peptide in human fetuses with growth restriction. Ultrasound Obstet Gynecol 29: 296–303. 17323307 10.1002/uog.3934

[12] Crispi F, Hernandez-Andrade E, Pelsers M, Plasencia W, Benavides-Serralde JA, et al (2008) Cardiac dysfunction and cell damage across clinical stages of severity in growth-restricted fetuses. Am J Obstet Gynecol 199: 254.e1–254.e8. 18771973 10.1016/j.ajog.2008.06.056

[13] Kocylowski RD, Dubiel M, Gudmundsson S, Sieg I, Fritzner E, et al (2009) Biochemical tissue-specific injury markers of the heart and brain in postpartum cord blood. Am J Obstet Gynecol 200: 273.e1–273.e25. 19167692 10.1016/j.ajog.2008.10.009

[14] Lechner E, Wiesinger-Eidenberger G, Wagner O, Weissensteiner M, Schreier-Lechner E, et al (2009) Amino terminal pro B-type natriuretic peptide levels are elevated in the cord blood of neonates with congenital heart defect. Pediatr Res 66: 466–469. 19581836 10.1203/PDR.0b013e3181b3aee4

[15] Merz WM, Kübler K, Albers E, Stoffel-Wagner B, Gembruch U (2012) N-terminal pro-B-type natriuretic peptide in the circulation of fetuses with cardiac malformations. Clin Res Cardiol 101: 73–79. 21960417 10.1007/s00392-011-0366-4

[16] Merz WM, Kübler K, Fimmers R, Stoffel-Wagner B, Geipel A, et al (2012) Circulating n-terminal pro-B-type natriuretic peptide in fetal anemia before and after treatment. Ped Res 72: 174–178. 10.1038/pr.2012.53 22546865

[17] Mari G, Deter RL, Carpenter RL, Rahman F, Zimmerman R, et al (2000) Noninvasive diagnosis by Doppler ultrasonography of fetal anemia due to maternal red-cell alloimmunization. Collaborative Group for Doppler Assessment of the Blood Velocity in Anemic Fetuses. N Engl J Med 342: 9–14. 10620643 10.1056/NEJM200001063420102

[18] Firpo C, Hoffman JIE, Silverman NH (2001) Evaluation of fetal heart dimensions from 12 weeks to term. Am J Cardiol 87: 594–600. 11230845 10.1016/s0002-9149(00)01437-5

[19] Merz WM, Kübler K, Albers E, Stoffel-Wagner B, Gembruch U (2010) Reference values for N-terminal pro-B-type natriuretic peptide in fetal circulation between 20 and 34 weeks of gestation. Clin Biochem 43: 519–521. 19968983 10.1016/j.clinbiochem.2009.11.012

[20] Bökenkamp A, Dieterich C, Dressler F, Mühlhaus K, Gembruch U, et al (2001) Fetal serum concentrations of cystatin C and ß_2_-microglobulin as predictors of postnatal kidney function. Am J Obstet Gynecol 185: 468–475. 11518911 10.1067/mob.2001.115283

[21] Hammerer-Lercher A, Mair J, Tews G, Puschendorf B, Sommer R (2005) N-terminal pro-B-type natriuretic peptide concentrations are markedly higher in the umbilical cord blood of newborns than in their mothers. Clin Chem 51: 913–915. 15855671 10.1373/clinchem.2004.046557

[22] Saito Y (2010) Roles of atrial natriuretic peptide and its therapeutic use. J Cardiol 56: 262–70. 20884176 10.1016/j.jjcc.2010.08.001

[23] Ordonez-Llanos J, Collinson PO, Christenson RH (2008) Amino-terminal pro-B-type natriuretic peptide: Analytic considerations. Am J Cardiol 101(suppl): 9A–15A. 18243867 10.1016/j.amjcard.2007.11.013

[24] Sivasankaran S, Sharland GK, Simpson JM (2005) Dilated cardiomyopathy presenting during fetal life. Cardiol Young 15: 409–416. 16014190 10.1017/S1047951105000855

[25] Pedra SRFF, Smallhorn JF, Ryan G, Chitayat D, Taylor GP, et al (2002) Fetal cardiomyopathies: Pathogenic mechanisms, hemodynamic findings, and clinical outcome. Circulation 106: 585–591. 12147541 10.1161/01.cir.0000023900.58293.fe

[26] Veniant M, Menard J, Bruneval P, Morley S, Gonzales MF, et al (1996) Vascular damage without hypertension in transgenic rats expressing prorenin exclusively in the liver. J Clin Invest 98: 1966–1970. 8903314 10.1172/JCI119000 PMC507639

[27] Saris JJ, ‘t Hoen PAC, Garrelds IM, Dekkers DHW, den Dunnen JT, et al (2006) Prorenin induces intracellular signalling in cardiomyocytes independently of angiontensin II. Hypertension 48: 564–571. 16940215 10.1161/01.HYP.0000240064.19301.1b

[28] deFilippi CR, Christenson RH (2009) B-type natriuretic peptide (BNP)/nt-proBNP and renal function: Is the controversy over? Clin Chem 55: 1271–1273. 19460836 10.1373/clinchem.2009.128157

[29] Yamashita T, Seino Y, Ogawa A, Ogata K-I, Fukushima M, et al (2010) N-terminal pro-BNP is a novel biomarker for integrated cardio-renal burden and early risk stratification in patients admitted for cardiac emergency. J Cardiol 55: 377–383. 20350516 10.1016/j.jjcc.2010.01.008

[30] Rinat C, Becker-Cohen R, Nir A, Feinstein S, Algur N, et al (2012) B-type natriuretic peptides are reliable markers of cardiac strain in CKD pediatric patients. Pediatr Nephrol 27: 617–625. 22038201 10.1007/s00467-011-2025-4

[31] Rychik J, McCann M, Tian Z, Bebbington M, Johnson MP (2010) Fetal cardiovascular effects of lower urinary tract obstruction with giant bladder. Ultrasound Obstet Gynecol 36: 682–686. 20503245 10.1002/uog.7664

[32] Gibson KJ, Lumbers ER (1999) Effects of bilateral nephrectomy and angiotensin II replacement on body fluids in foetal sheep. Clin Exp Pharmacol Physiol 26: 765–773. 10549399 10.1046/j.1440-1681.1999.03127.x

[33] Sanjuan R, Martin Oliva S, Blasco ML, Puchades M, Torregrosa I, et al (2011) Plasma brain natriuretic peptide levels in cardiac function assessment in chronic dialysis patients. Cardiorenal Med 1: 147–155. 22258537 10.1159/000329337 PMC3130986

[34] Nybo M, Benn M, Mogelvang R, Jensen JS, Schnohr P, et al (2007) Impact of hemoglobin on plasma proB-type natriuretic peptide concentrations in the general population. Clin Chem 53: 1921–1927. 17872941 10.1373/clinchem.2007.089391

[35] Berry SM, Lecolier B, Smith RS, Bercau G, Dombrowski MP, et al (1995) Predictive value of fetal serum ß_2_-microglobulin for neonatal renal function. Lancet 345 (8960): 1277–1278. 10.1016/s0140-6736(95)90928-1 7746060

[36] deVore GR (2005) The use of Z-scores in the analysis of fetal cardiac dimensions. Ultrasound Obstet Gynecol 26: 596–598. 16254876 10.1002/uog.2605

